# Comparative speed of kill of sarolaner (Simparica™ Chewables) and fluralaner (Bravecto^®^) against induced infestations of *Amblyomma americanum* on dogs

**DOI:** 10.1186/s13071-016-1684-1

**Published:** 2016-07-18

**Authors:** Robert H. Six, David R. Young, Melanie R. Myers, Sean P. Mahabir

**Affiliations:** Zoetis, Veterinary Medicine Research and Development, 333 Portage St., Kalamazoo, MI 49007 USA; YVRS, 7243 East Ave, Turlock, CA 95380 USA

**Keywords:** *Amblyomma americanum*, Tick, Dog, Simparica™, Sarolaner, Bravecto^®^, Fluralaner, Isoxazoline, Oral, Speed of kill

## Abstract

**Background:**

The lone star tick*, Amblyomma americanum*, infests dogs and cats in North America and transmits the pathogens *Ehrlichia chaffeensis* and *Ehrlichia ewingii*, which cause monocytic and granulocytic ehrlichiosis in dogs and humans, and *Cytauxzoon felis* which causes cytauxzoonosis in cats. A parasiticide’s speed of kill is important to minimize the direct deleterious effects [related to blood-feeding] of tick infestation and reduce the risk of transmission of tick-borne pathogens. In this study the speed of kill of sarolaner (Simparica™ Chewables) administered monthly for 3 months against *A. americanum* on dogs was evaluated and compared with a single dose of fluralaner (Bravecto^®^) for 13 weeks.

**Methods:**

Based on pretreatment tick counts, 24 dogs were randomly allocated to treatment with placebo or sarolaner at the label rate (2 to 4 mg/kg) on Days 0, 30 and 60 or with fluralaner (25 to 56 mg/kg) once according to manufacturer’s instructions on Day 0. Dogs were examined and live ticks counted at 8, 12, and 24 h after treatment and subsequent re-infestations on Days 14, 28, 42, 58, 76 and 90. Acaricidal efficacy was determined at each time point relative to counts for placebo dogs.

**Results:**

Monthly oral doses of sarolaner provided > 95 % efficacy within 24 h of treatment, and consistently provided > 70 % efficacy against subsequent re-infestations with ticks within 24 h over the entire treatment period. Significantly more live ticks were recovered from fluralaner-treated dogs than from sarolaner-treated dogs at 24 h after re-infestation from Day 42 onwards. At 24 h, efficacy of fluralaner was ≤ 20 % from Day 42 to the end of the study on Day 90. There were no adverse reactions to treatment.

**Conclusions:**

In this controlled laboratory evaluation, monthly treatment with sarolaner provided consistent efficacy against *A. americanum* with > 70 % of ticks killed within 24 h after a single oral dose over the duration of the study. Monthly treatment with sarolaner consistently killed significantly more ticks within 24 h than a single dose of fluralaner from 6 weeks after initial treatment.

## Background

Pet owners and veterinarians recognize ticks to be both a nuisance and a threat to animal and human health; infestations cause direct damage as a result of tick feeding behavior, and ticks transmit pathogens of veterinary and zoonotic importance [1]. The lone star tick, *Amblyomma americanum,* can bite humans, livestock, dogs and cats as well as many other mammals, and is one of the most common tick species infesting dogs in North America [2]. Its geographic range has expanded from the southern states [3], across the southern plains, through the Midwest and into the eastern states. Focal populations have also been reported in northern states including Maine, New York, Massachusetts, Connecticut and New Jersey [4, 5]. *Amblyomma americanum* transmits *Ehrlichia chaffeensis* and *Ehrlichia ewingii* [2, 6] to dogs and humans, which cause monocytic and granulocytic ehrlichiosis, respectively, and *Borrelia lonestari*, a probable cause of erythema migrans in humans, has also been detected in *A. americanum* using DNA amplification techniques [7].

Tick prevention and control have taken on a new importance as the awareness of, and exposure to, tick-borne diseases increases and *A. americanum* (and other tick species) populations expand. Topically administered parasiticides with contact activity have traditionally been the most commonly used approach for tick control on dogs, but recently a new class of compounds, the isoxazolines, have demonstrated efficacy against ticks for 1 month or longer following a single oral dose [8–10]. Sarolaner has demonstrated > 95 % efficacy against *A. americanum* within 48 h after re-infestation for 35 days [10], while afoxolaner has been reported to provide ≥ 98.9 % efficacy against *A. americanum* for up to 30 days after a single dose when assessed at 72 h after infestation [11]. Another isoxazoline, fluralaner, has demonstrated > 90 % efficacy against *A. americanum* for only 8 weeks when assessed at 72 h after infestation [12].

The speed of acaricidal activity is critical in disrupting or preventing feeding of ticks, thus reducing the risk of pathogen transmission. While pathogen transmission generally occurs after the infected tick is attached and feeds for at least 24 to 48 h [13, 14], transmission of *Ehrlichia canis* by *Rhipicephalus sanguineus* has recently been shown to occur within as little as 3 h after attachment under laboratory conditions [15]. Sarolaner in a chewable tablet formulation (Simparica™ Chewables) provides control of fleas and ticks for at least 1 month after a single oral dose [16]. Here we report a laboratory study conducted to evaluate and compare the speed of kill of a monthly oral dose of sarolaner with a single oral dose of fluralaner (Bravecto^®^) against an existing infestation and against re-infestations with *A. americanum* over a period of 90 days.

## Methods

The study was a masked, negative controlled, randomized laboratory comparative efficacy study conducted in California, USA. Study procedures were in accordance with the World Association for the Advancement of Veterinary Parasitology (WAAVP) guidelines for evaluating the efficacy of parasiticides for the treatment, prevention and control of flea and tick infestation on dogs and cats [17], and complied with the principles of Good Clinical Practices [18]. Masking of the study was assured through the separation of functions. All personnel conducting observations or animal care or performing infestations and counts were masked to treatment allocation.

### Animals

Thirteen male and 11 female, purpose-bred Beagles from 34 to 58 months of age and weighing from 8.6 to 13.2 kg were included in the study. Each dog was individually identified by a unique ear tattoo or electronic transponder and had undergone an adequate wash-out period to ensure that no residual ectoparasiticide efficacy remained from any previously administered treatments as demonstrated by live tick retention at the host suitability evaluation. Dogs were individually housed in indoor runs that conformed to accepted animal welfare guidelines and ensured no direct contact between dogs. Dogs were acclimatized to these conditions for at least 14 days prior to treatment. Dogs were fed an appropriate maintenance ration of a commercial dry canine feed for the duration of the study. Water was available *ad libitum*. All dogs were given a physical exam to ensure that they were in good health at enrollment and suitable for inclusion in the study. General health observations were performed at least once daily throughout the study.

### Design

The study followed a randomized complete block design. Twenty four dogs were ranked according to decreasing tick counts into blocks of 3, and within each block dogs were randomly allocated to treatment with placebo, sarolaner, or fluralaner, resulting in 8 dogs in each treatment group. Tick counts were conducted at 8, 12 and 24 h after treatment and each subsequent re-infestation.

### Treatment

On Days 0, 30 and 60, two groups of dogs each received either a placebo tablet or an appropriate Simparica™ Chewable tablet to provide the label dose (sarolaner at 2 to 4 mg/kg). The third group of dogs received a Bravecto^®^ tablet (per label providing fluralaner at 25 to 56 mg/kg) on Day 0 and placebo tablets on Days 30 and 60. All dogs were fed their regular ration within 30 min prior to treatment administration on Day 0 in order to comply with Bravecto^®^ label directions. The tablet(s) were administered by hand pilling to ensure accurate and complete dosing. Each dog was observed for several minutes after dosing for evidence that the dose was swallowed, and for general health at 1, 4, and 24 h after treatment administration, and at least once daily for the duration of the study. Dogs were observed approximately two hours after dosing for evidence of emesis.

### Tick infestation and assessment

The ticks were obtained from the Oklahoma State University’s *A. americanum* colony, which was initiated in 1976 with engorged females collected locally in Stillwater, OK. The colony has been maintained with the introduction of locally collected, engorged females every 2 years. The most recent introduction was approximately 4 months before the study was initiated. Ticks were considered pathogen free, as the unfed adult ticks used in this study had been maintained for at least one generation in the colony on pathogen-free hosts.

Tick infestations were performed on Days -9 (host suitability and allocation), -2, 14, 28, 42, 58, 76 and 90. For each infestation dogs were sedated with a combination of ketamine and xylazine, a pre-counted aliquot of 50 (±5; 1:1 sex ratio) viable unfed adult *A. americanum* was directly applied to the dog, which was then confined in an appropriately sized travel crate for approximately 4 h to restrict movement and facilitate tick attachment. Each dog was examined to remove and count live ticks at 48 h after the initial host suitability infestation. At 8 and 12 (±1) hours after treatment and each subsequent re-infestation, the dogs were examined systematically so that the entire body surface was carefully examined and live ticks were counted *in situ*. At 24 h after treatment and each subsequent re-infestation, the dogs were examined and then thoroughly combed to count and remove live ticks. Each dog was examined for at least 10 min. If ticks were encountered in the last minute, combing was continued in 1 min increments until no ticks were encountered.

### Statistical analysis

The individual dog was the experimental unit and the primary endpoint was the live tick count. Data for post-treatment live (free plus attached) tick counts were summarized with arithmetic (AM) and geometric (GM) means by treatment group and time point. Tick counts were transformed by the log_e_(count + 1) transformation prior to analysis in order to stabilize the variance and normalize the data. Using the PROC MIXED procedure (SAS 9.2, Cary NC), transformed counts were analyzed using a mixed linear model. The fixed effects were treatment, time point and the interaction between time point and treatment by time point. The random effects included block, block by treatment interaction and error. Testing was two-sided at the significance level α = 0.05.

The assessment of efficacy for live ticks was based on the percent reduction in the arithmetic and geometric mean live tick counts relative to placebo and to the positive control, as suggested by the most recent guidelines of the WAAVP for systemic acaricides [17] and was calculated using Abbott’s formula:

$$ \%\ \mathrm{reduction}=100 \times \frac{\mathrm{mean}\ \mathrm{count}\ \left(\mathrm{placebo}\right)\hbox{--} \mathrm{mean}\ \mathrm{count}\ \left(\mathrm{treated}\right)}{\mathrm{mean}\ \mathrm{count}\ \left(\mathrm{placebo}\right)} $$

## Results

There were no treatment-related adverse events during the study. Placebo-treated dogs maintained tick infestations throughout the study with mean tick counts ranging from approximately 10 to 25 (Tables 1, 2 and 3).

**Table 1:** Mean live *Amblyomma americanum* counts and efficacy relative to placebo at 8 hours after treatment and post-treatment re-infestations for dogs treated with oral sarolaner on Days 0, 30, and 60 or fluralaner once on Day 0

Treatment		Day of treatment or re-infestation						
		0	14	28	42	58	76	90
Placebo	Range	17–31	4–18	7–24	9–28	9–30	8–26	7–23
	A. mean	23.5	11.6	15.1	15.8	19.9	16.1	17.5
	G. mean^1^	23.1^a^	10.6^a^	13.6^a^	14.6^a^	18.8^a^	15.0^a^	16.6^a^
Sarolaner	Range	6–28	2–15	7–20	9–34	6–18	3–22	10–24
	A. mean	16.8	9.3	12.0	17.3	12.8	14.4	16.1
	Efficacy (%)	28.7	20.4	20.7	0.0	35.8	10.9	7.9
	G. mean^1^	15.4^a,b^	7.8^a^	11.4^a^	15.9^a^	12.3^a^	12.7^a^	15.5^a^
	Efficacy (%)	33.2	26.3	16.1	0.0	34.7	15.3	6.7
	*P*-value *vs* placebo	0.1338	0.2817	0.5240	0.7543	0.1177	0.5432	0.7973
Fluralaner	Range	2–30	2–17	8–18	5–26	12–27	7–19	11–30
	A. mean	16.3	8.1	14.1	19.5	16.6	13.3	17.0
	Efficacy (%)	30.9	30.1	6.6	0.0	16.4	17.8	2.9
	G. mean^1^	12.7^b^	7.0^a^	13.6^a^	17.7^a^	16.1^a^	12.7^a^	16.2^a^
	Efficacy (%)	45.0	33.8	0.4	0.0	14.0	15.7	2.6
	*P*-value *vs* placebo	0.0218	0.1317	0.9883	0.4619	0.5580	0.5149	0.9203
	*P*-value *vs* sarolaner	0.5337	0.7457	0.5867	0.7321	0.3792	0.9889	0.8883

^1^Geometric means within columns with the same superscript are not significantly different (*P* > 0.05)

**Table 2:** Mean live *Amblyomma americanum* counts and efficacy relative to placebo at 12 hours after treatment and post-treatment re-infestations for dogs treated with oral sarolaner on Days 0, 30, and 60 or fluralaner once on Day 0

Treatment		Day of treatment or re-infestation						
		0	14	28	42	58	76	90
Placebo	Range	18–32	3–17	7–23	3–27	6–28	7–27	7–25
	A. mean	24.1	10.5	15.1	14.3	18.4	14.5	17.3
	G. mean^1^	23.8^a^	9.3^a^	13.9^a^	12.4^a^	17.2^a^	13.0^a^	16.4^a^
Sarolaner	Range	2–15	2–14	8–20	6–24	7–16	3–22	9–23
	A. mean	7.3	7.8	11.9	12.6	11.3	11.9	13.9
	Efficacy (%)	69.9	26.2	21.5	11.4	38.8	18.1	19.6
	G. mean^1^	5.7^c^	6.7^a,b^	11.3^a^	11.5^a^	10.9^a^	9.8^a^	13.2^a^
	Efficacy (%)	76.0	27.5	18.3	7.5	36.6	24.6	19.0
	*P*-value *vs* placebo	< 0.0001	0.2624	0.4612	0.7786	0.0972	0.3085	0.4375
Fluralaner	Range	3–29	0–14	2–19	5–36	15–17	5–19	11–28
	A. mean	13.5	5.1	10.5	21.3	15.8	12.0	16.4
	Efficacy (%)	44.0	51.2	30.6	0.0	14.3	17.2	5.1
	G. mean^1^	11.1^b^	3.6^b^	8.9^a^	19.1^a^	15.7^a^	11.0^a^	15.6^a^
	Efficacy (%)	53.4	61.4	35.9	0.0	8.7	15.6	4.8
	*P*-value *vs* placebo	0.0038	0.0012	0.0966	0.1010	0.7241	0.5211	0.8494
	*P*-value *vs* sarolaner	0.0440	0.0750	0.4523	0.1060	0.2476	0.7240	0.6037

^1^Geometric means within columns with the same superscript are not significantly different (*P* > 0.05)

**Table 3:** Mean live *Amblyomma americanum* counts and efficacy relative to placebo at 24 hours after treatment and post-treatment re-infestations for dogs with oral sarolaner on Days 0, 30, and 60 or fluralaner once on Day 0

Treatment		Day of treatment or re-infestation						
		0	14	28	42	58	76	90
Placebo	Range	19–31	3–18	8–21	7–37	10–32	6–26	8–21
	A. mean	24.6	10.8	13.1	17.4	19.8	14.9	16.5
	G. mean^1^	24.4^a^	9.4^a^	12.6^a^	15.5^a^	18.7^a^	13.4^a^	15.7^a^
Sarolaner	Range	0–7	0–2	0–10	0–10	2–14	0–5	0–9
	A. mean	0.9	1.0	3.6	2.8	6.1	1.1	2.6
	Efficacy (%)	96.4	90.7	72.4	84.2	69.0	92.4	84.1
	G. mean^1^	0.3^b^	0.8^b^	2.1^b^	1.3^b^	5.4^b^	0.8^b^	1.7^b^
	Efficacy (%)	98.8	91.6	83.1	91.6	71.3	94.3	89.4
	*P*-value *vs* placebo	< 0.0001	< 0.0001	< 0.0001	< 0.0001	< 0.0001	< 0.0001	< 0.0001
Fluralaner	Range	0–4	0–9	0–11	6–32	13–24	7–18	7–26
	A. mean	0.9	2.6	3.4	19.1	16.9	11.6	15.9
	Efficacy (%)	96.4	75.6	74.3	0.0	14.6	21.8	3.8
	G. mean^1^	0.5^b^	1.4^b^	2.2^b^	17.4^a^	16.6^a^	11.2^a^	14.8^a^
	Efficacy (%)	97.8	85.2	82.4	0.0	11.5	16.8	5.8
	*P*-value *vs* placebo	< 0.0001	< 0.0001	< 0.0001	0.6487	0.6360	0.4870	0.8182
	*P*-value *vs* sarolaner	0.5715	0.3246	0.9247	< 0.0001	0.0006	< 0.0001	< 0.0001

^1^Geometric means within columns with the same superscript are not significantly different (*P* > 0.05)

At the 8-h time point, tick counts for both products were not significantly different from placebo-treated dogs or each other (*P* ≥ 0.1177) at any evaluation except on Day 0 when counts for the fluralaner-treated dogs were significantly lower than placebo (*P* = 0.0218) but no different to those for sarolaner treated dogs (*P* = 0.5337). Efficacy at Day 0 was 45.0 % for fluralaner and 33.2 % for sarolaner and subsequent percent reductions ranged from 0 to 33.8 % for fluralaner and 0 to 34.7 % for sarolaner (GM) (Table 1).

At the 12-h time point, sarolaner-treated dogs had significantly lower tick counts than placebo-treated dogs (*P* < 0.0001) on Day 0 with efficacy (GM) of 76.0 % (Table 2). Treatment with fluralaner resulted in significantly lower tick counts than placebo at 12 h on Days 0 and 14 (*P* ≤ 0.0038) with efficacies (GM) of 53.4 and 61.4 %, respectively. Tick counts for sarolaner were significantly lower than for fluralaner on Day 0 (*P* = 0.0440) and similar on Day 14 (*P* = 0.0750) with efficacies of 76.0 and 27.5 % respectively. The tick counts were similar for placebo, sarolaner and fluralaner-treated dogs on all other count days (*P* ≥ 0.0966) and percent reductions ranged from 0.0 to 35.9 % for fluralaner and 7.5 to 36.6 % for sarolaner (GM) (Table 2).

**Fig. 1:**
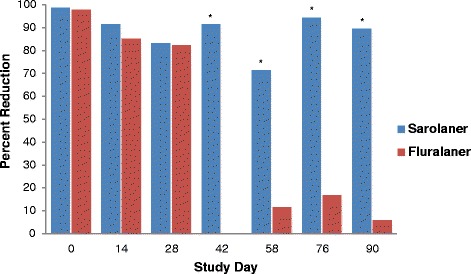
Percent efficacy based on geometric mean counts relative to placebo at 24 hours after treatment and weekly post-treatment re-infestations of *Amblyomma americanum* for dogs treated with oral sarolaner on Days 0, 30, and 60 or fluralaner once on Day 0. *Geometric mean live tick counts for sarolaner significantly lower than those for fluralaner (*P*≤ 0.05)

At the 24-h time point, dogs treated with sarolaner had significantly lower tick counts than placebo-treated dogs (*P* < 0.0001) on all count days, and these counts were also lower than those for fluralaner-treated dogs (*P* ≤ 0.0006) from Day 42 to Day 90 (Table 3). Fluralaner-treated dogs had significantly lower tick counts than placebo (*P* < 0.0001) from Day 0 to Day 28 only. Treatment with sarolaner resulted in efficacy of at least 71.3 % (GM) to Day 90, while efficacy for dogs treated with fluralaner declined below 20 % (GM) and was no different from placebo (*P* ≥ 0.4870) from Day 42 to Day 90 (Table 3, Fig. 1).

## Discussion

Sarolaner provided a significant reduction in *A. americanum* tick counts within 12 h after the first treatment on Day 0 and demonstrated consistently high efficacy of > 70 % (GM) within 24 h after re-infestations over the three month (three dose) period. In contrast, a single dose of fluralaner only provided significant reductions in tick counts through Day 28 when assessed up to 24 h after infestation and counts at 24 h were no different to placebo from Day 42 onwards. Thus, monthly treatment with sarolaner resulted in a consistent speed of kill of *A. americanum* ticks over the three month period, while the speed of kill of fluralaner was significantly lower from Day 42 with counts no different from placebo at 24 h after infestation. These results are consistent with the reported performance of the two products; sarolaner provides efficacy ≥ 96.5 % within 48 h for 35 days after a single dose against five tick species [10] and ≥ 89.6 % efficacy against re-infestations of *Amblyomma maculatum* within 24 h for 35 days [19]. Fluralaner has efficacy against *Dermacentor variabilis*, *Ixodes scapularis* and *R. sanguineus* for up to 12 weeks after a single dose based on counts at 48 h, but only 8 weeks of persistent efficacy versus *A. americanum* when assessed at 72 h after infestation [12]. These results also concur with a previous study in which the speed of kill of fluralaner against *R. sanguineus* and *Dermacentor reticulatus* similarly decreased in the third month after treatment [20].

On Day 42, the efficacy of sarolaner and flurolaner at the 8 and 12 h time points ranged from 0.0 to 7.5 % (GM). The reason for this apparent lower efficacy of both products relative to the same time points on other Days is unknown. Because the effect was observed with both products, and the tick counts on the placebo-treated dogs were generally consistent with those from other post-treatment infestations, it is plausible that there was a relatively prolonged period of time between tick infestation and the initiation of tick feeding, which resulted in delayed drug exposure. This can happen if ticks are not held for sufficient time after molting from the previous stage to allow optimal priming for feeding. This results in ticks questing longer and taking additional time to attach and commence blood feeding.

The perceived benefit of a longer treatment interval (such as 8 to 12 weeks for fluralaner) and hence the need for less treatments, needs to be balanced with the potential risk of an unpredictable decline in efficacy at the end of the claimed treatment period. Also, the variability in efficacy and the duration of the period of protection provided by fluralaner against different tick genera and species could be confusing for prescribers and users who may not be familiar with the ticks in their region. An important benefit of monthly sarolaner administration is that it provides sustained efficacy against relevant tick species in dogs and maintains a consistent speed of kill for the entire duration of the month. Rapid and consistent kill of ticks is important to reduce the direct adverse effects of tick feeding and is critical in the reduction of tick-borne pathogen transmission. Thus, the consistent efficacy of a single oral dose of sarolaner shown in this study should help to reduce the risk of a treated dog on a monthly treatment regime becoming infected with the pathogens transmitted by *A. americanum*. Further studies directly examining the effect of treatment on the transmission and infectivity of these individual pathogens are needed to confirm the levels of protection provided through sarolaner’s acaricidal efficacy.

## Conclusions

This study confirmed the consistent acaricidal efficacy of monthly treatment with sarolaner against *A. americanum* and demonstrated that >70 % of ticks were killed within 24 h during the entire treatment period. Monthly treatment with sarolaner killed significantly more ticks within 24 h than a single dose of fluralaner from 6 weeks after initial treatment.

## Abbreviations

Not applicable
